# Dye amount quantification of Papanicolaou-stained cytological images by multispectral unmixing: spectral analysis of cytoplasmic mucin

**DOI:** 10.1117/1.JMI.12.1.017501

**Published:** 2024-12-28

**Authors:** Saori Takeyama, Tomoaki Watanabe, Nanxin Gong, Masahiro Yamaguchi, Takumi Urata, Fumikazu Kimura, Keiko Ishii

**Affiliations:** a Tokyo Institute of Technology, School of Engineering, Department of Information and Communications Engineering, Yokohama, Japan; b Shinshu University, School of Health Sciences, Department of Biomedical Laboratory Sciences, Matsumoto, Japan; c Okaya City Hospital, Division of Diagnostic Pathology, Okaya, Japan

**Keywords:** dye amount quantification, color unmixing, multispectral image, Papanicolaou stain, Lobular endocervical glandular hyperplasia detection

## Abstract

**Purpose:**

The color of Papanicolaou-stained specimens is a crucial feature in cytology diagnosis. However, the quantification of color using digital images is challenging due to the variations in the staining process and characteristics of imaging equipment. The dye amount estimation of stained specimens is helpful for quantitatively interpreting the color based on a physical model. It has been realized with color unmixing and applied to staining with three or fewer dyes. Nevertheless, the Papanicolaou stain comprises five dyes. Thus, we employ multispectral imaging with more channels for quantitative analysis of the Papanicolaou-stained cervical cytology samples.

**Approach:**

We estimate the dye amount map from a 14-band multispectral observation capturing a Papanicolaou-stained specimen using the actual measured spectral characteristics of the single-stained samples. The estimated dye amount maps were employed for the quantitative interpretation of the color of cytoplasmic mucin of lobular endocervical glandular hyperplasia (LEGH) and normal endocervical (EC) cells in a uterine cervical lesion.

**Results:**

We demonstrated the dye amount estimation performance of the proposed method using single-stain images and Papanicolaou-stain images. Moreover, the yellowish color in the LEGH cells is found to be interpreted with more orange G (OG) and less Eosin Y (EY) dye amounts. We also elucidated that LEGH and EC cells could be classified using linear classifiers from the dye amount.

**Conclusions:**

Multispectral imaging enables the quantitative analysis of dye amount maps of Papanicolaou-stained cytology specimens. The effectiveness is demonstrated in interpreting and classifying the cytoplasmic mucin of EC and LEGH cells in cervical cytology.

## 1 Introduction

Pathological diagnosis is a field of medicine in which specimens prepared from disease tissues are observed under a microscope to morphologically assess the disease, thereby revealing its cause, mechanism, and progression. It is an essential technique to detect tumors and distinguish whether they are benign or malignant tumors. In pathological diagnosis, a histological specimen collected from a patient is stained, and pathologists observe the tissue or cells using an optical microscope.

Recently, the whole slide imaging technology has matured,^1^ enabling the capture of specimens as high-resolution digital images. Adopting various image processing techniques is expected to deliver quantitative results, leading to more accurate and less subjective diagnoses, thereby providing significant benefits. Cytopathology is a division of pathology in which cytological specimens are examined under a microscope to detect abnormal cells for a pathological or clinical diagnosis. Uterine cervical cytology, also known as the Pap smear test, is a significant field within cytopathology. Cells of the cervix are scraped, and a cytological specimen is prepared using Papanicolaou staining for microscopic examination. In cervical cancer diagnosis, one type of the benign lesion, lobular endocervical glandular hyperplasia (LEGH), is believed to be a precancerous lesion of minimal deviation adenocarcinoma (MDA) and adenocarcinoma of gastric types in the uterine cervix.^2^ The visual identification of cells that comprise LEGH from normal endocervical (EC) cells is often difficult as the nuclear atypia is not significant in LEGH cells.^3^ In Ref. 4, the authors revealed the characteristics of the nuclei of EC and LEGH cells, where EC cell nuclei are larger than LEGH cell nuclei, and nuclei of LEGH cells have irregular nuclear membrane structures and an elongated shape, and demonstrated that the classification performance using only the characteristics is ∼80% to 90 %. On the other hand, Ishii et al.^5^^,^^6^ indicated that mucinous MDA and gastric metaplastic lesion (i.e., LEGH) have cytoplasmic mucins stained yellow/orange with Papanicolaou staining. In contrast, EC cells have acid mucin appearing pink, and a distinctive two-color pattern is an important clue for the identification of LEGH cells. However, there are no quantitative diagnostic criteria based on the color of the mucin in the cervical cell specimen.

For quantification of color in the Papanicolaou-stained cytological image, some methods are proposed,^7^^,^^8^ which reveal the relationship between dyes and the RGB (red, green, and blue) values. However, as the RGB values captured by a color camera are device-dependent, the methods cannot be a true quantification of the Papanicolaou stain. The method proposed in Ref. 9 uses the standardized color space, CIELAB, by applying a color calibration procedure, but the color is also affected by the staining process, such as the staining time and the recipe of the staining agent. Instead of using a color space based on a psychophysical quantity, a physical quantity, the abundance of dye, can also be employed for color quantification. In Papanicolaou staining, the cell nucleus and phospholipid are stained with hematoxylin (H) and bismarck brown Y (BY), respectively. The cytoplasm is stained with eosin Y (EY), light green SF (LG), or orange G (OG), with strengths that depend on factors such as molecular weight, diffusibility, and differentiation. Providing a quantitative description based on the dye abundance (or amount of each dye) is advantageous for interpreting the physical phenomenon behind the stained sample.

Dye amount estimation, or color unmixing, has been employed in histopathology image analysis.^10^ For example, in hematoxylin and eosin (H&E) staining, a color image is decomposed into H and E component images, where the H component image can be used for cell nucleus segmentation. In immunohistochemistry (IHC), color unmixing separates 3,3′-Diaminobenzidine (DAB) and counterstains H components, and the DAB component image is often used to analyze the protein expression. Dye amount estimation has been implemented ordinarily using linear color unmixing or color deconvolution techniques. However, Papanicolaou staining uses five dyes, and linear color unmixing is impossible from RGB three-channel images as it becomes an ill-posed problem. This problem can be solved by multispectral (MS) unmixing,^11^^,^^12^ which involves linear unmixing with an MS image. Spectral unmixing (including MS unmixing) is mainly used to identify materials in a captured image in remote sensing and fluorescence imaging.^13^^,^^14^ The above applications target the reflected or emitted light from materials, whereas a Papanicolaou-stained cytology specimen is an absorption material, as is the H&E-stained histology specimen. In this case, the observation model needs to consider a nonlinear transform based on Lambert–Beer’s law, so it differs from the above ones. Image analysis techniques using spectral information have been actively studied in digital pathology. In Ref. 15, multiplex IHC was implemented using multispectral imaging for efficient image acquisition. Hyperspectral imaging has also been researched for legion detection in histology slides.^16^^,^^17^ However, there are no prior reports on applying MS unmixing to the quantification of dye amounts for classifying cytoplasmic mucin in cytology.

In this paper, we present the application of a dye amount estimation method based on MS unmixing to the cytology samples stained with Papanicolaou stain. For the proposed method, we first capture the single-stain samples using an MS camera to acquire the spectral absorption characteristics of all dyes required in MS unmixing. Thereby, the spectral absorption coefficient matrix of the Papanicolaou stain is obtained. Then, using the pseudo-inverse matrix, the proposed method enables estimating the amount of stain at every pixel in the microscopic image of the cytological specimen. In the experiments, we illustrate the performance feasibility of the proposed method by using single-stain and Papanicolaou-stained specimens. Moreover, we explore the potential of employing the proposed dye amount estimation technique to quantify mucin color and discern EC and LEGH cells, leveraging the estimated dye amount images. As a result, the yellowish mucin observed in LEGH is characterized as a composite of OG and LG; in particular, the increase in OG is the primary influence on the cytoplasmic mucin in LEGH. In our previous conference paper,^18^ we presented preliminary results of quantifying the color of mucin based on the dye amount estimation using an MS of the mucin region in the image, and this paper is the compilation of the whole study with additional details on the application of the method to the classification of EC and LEGH cells.

## 2 Materials and Methods

### 2.1 Specimens and Image Acquisition

Cytological samples of four cases were collected, three from the uterine cervix with conventional and one from the oral cavity with liquid-based cytology (LBC), for the experimental verification of the proposed dye amount estimation method depicted in Secs. 3.2 and 3.3. Then, we prepared six specimens from each case, a total of 24 specimens. One specimen from each case was dyed with Papanicolaou stain and the others with either EY, H, LG, OG, or BY. As a result, we can use six different specimens from each case. Moreover, four Papanicolaou-stained conventional specimens collected from a uterine cervix were used in the classification experiment of EC and LEGH cells presented in Secs. 3.4 and 3.5.

Papanicolaou staining was performed using Carrazzi’s hematoxylin solution (Hematoxylin, Sigma-Aldrich, St. Louis, Missouri, United States), OG-6 (Muto Pure Chemicals, Co. Ltd., Hongo, Tokyo), and EA-50 (Muto Pure Chemicals, Co. Ltd.). For single stain, LG, EY, and BY were used to create single-stain solutions of the same compositions as EA-50. The staining time is as follows: Carrazzi’s hematoxylin was used for 85 s and OG for 65 s, and the dyes included in EA-50 (LG, EY, and BY) were used for 170 s, respectively.

The images were collected with a 40× objective lens and an MS microscope (Vectra^®^3, PerkinElmer Inc., Waltham, Massachusetts, United States), which captured a 1020×1368×14 MS image with the spectral information from 440 to 720 nm at 20-nm intervals. The resolution of MS images was 0.25  μm/pixel. Section 3.1 describes the confirmation of the spectral characteristics of the MS microscope. We use in-house software on MATLAB for all image processing and analysis.

### 2.2 Color Unmixing of Papanicolaou-Stained Specimen

This work aims to estimate the dye amount in a Papanicolaou-stained specimen from an MS image. Our proposed method is based on color unmixing^10^ used in dye amount estimation methods for H&E and immunohistochemical (IHC) stains. By calculating the color transformation matrix, color unmixing estimates the abundance maps of the dyes from the observation. When the number of dyes in the stain is less than that of the channels of an observation, color unmixing can infer the dye amounts of each pixel from the observation. In contrast, when the number of dyes surpasses the number of channels, as seen in the case of capturing a Papanicolaou-stained specimen with RGB observations, the estimation of the dye amount map encounters challenges because of the underdetermined nature of the system. Based on the above discussion, the proposed method assumes that the observation is an MS image with over five bands.

### 2.3 Dye Amount Estimation from an MS Image

By assuming Lambert–Beer’s law, for a certain wavelength λ, the absorbance of an observation α(λ) is modeled by the following equations:

$$\alpha (\lambda )=\underset{i}{\sum }c_{i}\varepsilon_{i}(\lambda ),$$ (1)

$$I_{1}(\lambda )=10^{-\alpha (\lambda )}I_{0}(\lambda ),$$ (2)

where $c_{i}$ is the amount of an i’th stain (i=1,…,M, and M is the number of dyes), and $\varepsilon_{i}(\lambda )$ is the spectral absorption coefficient. Let $I_{0}(\lambda )$ and $I_{1}(\lambda )$ be the intensities of the incident and transmitted lights, which are obtained from the unstained (glass) and stained areas, respectively.

We consider a multichannel image capture, including RGB color imaging and MS imaging, which can be formulated as follows:

$$g_{k}=\int_{\lambda_{\min}}^{\lambda_{\max}}s_{k}(\lambda )I_{1}(\lambda )d\lambda ,\;k=1,\ldots ,N,$$

where $g_{k}$ and $s_{k}(\lambda )$ represent the pixel value of the captured image and the spectral sensitivity of the k’th band of the imaging system, respectively. Note that we assume a linear relationship between the light intensity and pixel value. The interval $\left[\lambda_{\min},\lambda_{\max}\right]$ denotes the wavelength range for the captured image. We also have

$$g_{0k}=\int_{\lambda_{\min}}^{\lambda_{\max}}s_{k}(\lambda )I_{0}(\lambda )d\lambda ,\;k=1,\ldots ,N.$$

According to Eq. (2), the spectral absorbance a(λ) can be calculated by

$$\alpha (\lambda )=\log(\frac{I_{0}(\lambda )}{I_{1}(\lambda )}).$$

However, we need to estimate $I_{1}(\lambda )$ and $I_{0}(\lambda )$ from the captured images $g_{k}$ and $g_{0k}$, respectively. As a rough approximation, if

$$s_{k}(\lambda )=\{\begin{array}{cc} 1, & \lambda =\lambda_{k},\\ 0, & \lambda \neq \lambda_{k}, \end{array}$$ (3)

we can define the spectral absorbance of the k’th channel $\alpha (\lambda_{k})$ as

$$\alpha (\lambda_{k})=\log(\frac{I_{0}(\lambda_{k})}{I_{1}(\lambda_{k})})=\log(\frac{g_{0k}}{g_{k}}).$$

The error in this assumption was analyzed in Ref. 19 and is not negligible when the number of bands is small, but most conventional techniques of color unmixing applied in pathology applications are based on this assumption.^10^ Then, we can reformulate Eq. (1) as

$$a=\mathrm{Hc}\;\mathrm{s.t.}\;a=(\alpha (\lambda_{1}),\alpha (\lambda_{2}),\ldots ,\alpha (\lambda_{N}){)}^{⊺}\in R^{N},\;H=[\begin{array}{cccc} \varepsilon_{1}(\lambda_{1}) & \varepsilon_{2}(\lambda_{1}) & \cdots & \varepsilon_{M}(\lambda_{1})\\ \varepsilon_{1}(\lambda_{2}) & \varepsilon_{2}(\lambda_{2}) & \cdots & \varepsilon_{M}(\lambda_{2})\\ \vdots & & \ddots & \vdots \\ \varepsilon_{1}(\lambda_{N}) & \varepsilon_{2}(\lambda_{N}) & \cdots & \varepsilon_{M}(\lambda_{N}) \end{array}]\in R^{N\times M},\;c=(c_{1},c_{2},\ldots ,c_{M}{)}^{⊺}\in R^{M}.$$ (4)

As M<N in the proposed method, we can estimate the dye amount vector c at each pixel by multiplying the pseudo-inverse of the spectral absorption coefficients matrix of dyes H with the absorbance vector a of the captured image. The matrix H is called the stain matrix hereafter for simplicity. In practice, we need to derive H experimentally because the color of the Papanicolaou stain depends on the recipe and the staining condition. Even when the spectral sensitivity of an imaging device does not satisfy the assumption outlined in Eq. (3), the linear model presented in Eq. (4) remains approximately valid by measuring the spectral absorption coefficients using the same MS imaging system with the narrowband spectral sensitivity. Moreover, if N is large, e.g., in MS and HS imaging cases, the linear model in Eq. (4) becomes more redundant, and a more robust estimation is possible by suppressing the characterization error. In this paper, we determine the stain matrix H based on Eq. (4) by capturing MS images of single-stain specimens, as explained in Sec. 2.4. There are existing methods to estimate the stain matrix H from the captured image, blind spectral unmixing or blind color deconvolution,^13^^,^^20^^,^^21^ which will be applied in the future for a more practical system.

### 2.4 Determination of the Stain Matrix H

To estimate the stain matrix H using the spectral absorption coefficients of dyes included in the Papanicolaou stain, we prepare single-stain specimens (EY, H, LG, OG, and BY) and capture them using the MS microscope. If the sample is stained with l’th dye only, $c=[c_{m}]$ in Eq. (3) can be written as

$$c_{m}=\{\begin{array}{cc} \beta & \text{if}\;m=l,\\ 0 & \text{otherwise}, \end{array}$$

where β denotes the abundance of the dye at each pixel. From the captured image, we have the spectral absorbance vector for the single-stain regions by l’th dye, denoted as $a_{l-\text{single}}$, which can be written as

$$a_{l-\text{single}}=\beta \varepsilon_{l},$$

where $\varepsilon_{l}=(\varepsilon_{l}(\lambda_{1}),\varepsilon_{l}(\lambda_{2}),\ldots ,\varepsilon_{l}(\lambda_{N}){)}^{⊺}$ represents the l’th column of the stain matrix H. As it is desirable to average the results of multiple pixels to reduce the effect of noise, we use

ε^l=average{al−single‖al−single‖22}

to estimate of the spectral absorption coefficient vector $\varepsilon_{l}$, and average{·} denotes the operator for averaging multiple pixels.

### 2.5 Normalization of Dye Amounts by Reference Values

For better interpretability of the quantified results, we normalize the estimated dye amount c, such that the dye amounts of the well-stained regions with dour stains become unit values. This is because each spectral absorption coefficient vector $\varepsilon_{l}$ is estimated from each single-stain image and represents a relative amount, not an absolute amount with the physical unit. Thanks to the normalization, the amount of H should be close to one within well-stained nuclei, and the cytoplasm of well-stained squamous cells should exhibit a unit value of one for EY, OG, or LG. For this purpose, the reference value of each dye is determined by selecting well-stained areas from the Papanicolaou-stained images, i.e., some nuclei for H, and cytoplasm regions for EY, OG, and LG. Then, we consider the almost maximum value of each dye amount as a reference value and obtain the normalized dye amount by dividing $c_{m}$ by the reference value.

### 2.6 Elimination of Background Regions

A specimen is generally on the glass, and the background is transparent. However, the background region of an actual stained specimen slide is slightly stained, as shown in Fig. 2, because washing after staining in each single stain process cannot completely remove the dye in the background glass region. Therefore, the thresholds for the background area, $T_{l}$, are determined from the histogram of the dye abundance. We regard the pixels in which the amount values of all dyes are less than the corresponding $T_{l}$ as non-stained pixels, namely, the background.

### 2.7 Classification of EC and LEGH Cells Based on the Dye Amounts in Mucinous Cytoplasm

In this paper, we explore the possibility of classifying EC and LEGH cells using the estimated dye amount map of endocervical cytology samples. First, we divide the map into patches of 10×10  pixels and extract the patches of cytoplasm regions. As the nucleus area is stained by H and the background area is unstained, the patches of the mucinous cytoplasm are separated from those of the cell nuclear area and background using the ratio of the stained pixels. Specifically, if the number of H-stained pixels is over 50%, i.e., 50 pixels, they are regarded as the cell nuclear patches. If the nonstained pixels occupy over b%, they are categorized as background patches. The threshold b needs to be adjusted using the image set.

For the classification of EC and LEGH mucin, we analyze the characteristics of the Papanicolaou stain and set the decision boundary. As it is preferable to interpret the reasoning for the classification, we use the decision boundary between EC and LEGH mucin in lower-dimensional space. Therefore, we devise the classification in the 2D space of EY and OG, and the 3D space of EY, LG, and OG. In the experimental analysis described in Sec. 3.4, it is confirmed that mucin is hardly stained by H, and there is a significant difference between EC and LEGH mucin in EY and OG amounts.

We adopt Fischer’s linear discriminant with EC and LEGH mucin patch samples to decide the boundary between EC and LEGH. The discriminant function D(c) takes the form

$$D(c)=w^{⊺}c,$$

where w is a linear transform vector, determined by the averages and the variance of the samples. After the coefficient vector w is determined, each patch can be classified into EC or LEGH class using D(c); if D≥0, it is an LEGH cell, and if D<0, it is an EC cell.

## 3 Experiment

### 3.1 Characterization of Microscopic Multispectral Imaging System

**Fig. 1:**
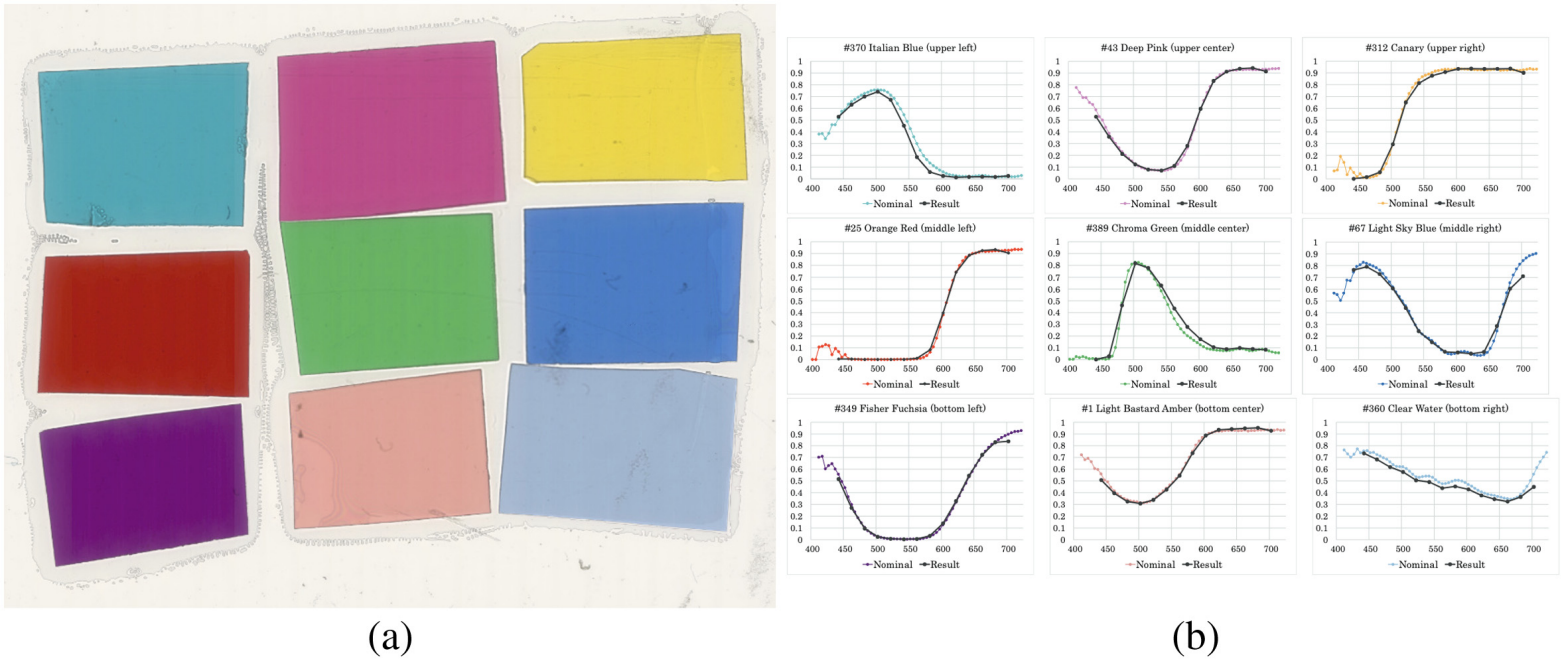
Color chart used for validating the spectral transmittance measurement by the MS imaging system (a) and the results (b).

In this experiment, we used an MS microscope PerkinElmer Vectra®3, which captures the spectral information from 440 to 720 nm at 20-nm intervals in 14 bands. First, we confirmed the spectral characteristics of the MS microscope by verifying that the spectral transmittance could be correctly derived by the 14-band MS image. For this purpose, we used the color chart slide,^22^^,^^23^ as shown in Fig. 1. The color chart consisted of nine miniature color filters (Roscolux, Rosco Lab., Stamford, Connecticut, United States) attached on a glass slide, which has been used for color calibration in digital pathology. The measured spectral transmittance data of nine color patches published in Ref. 23 were provided with the color chart.

**Fig. 2:**
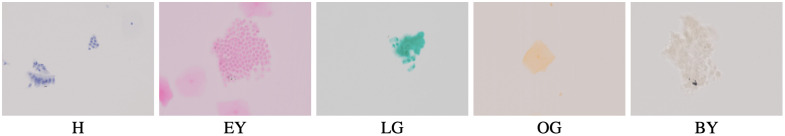
Examples of single-stain images used to calculate the spectral absorption coefficient matrix H.

We used Vectra^®^3 to capture the color chart and averaged the pixel values in a 200×200 area for each color patch, which provided $I_{1}(\lambda )$ in Eq. (2). The averaged pixel value was also calculated for the glass area in the same size, providing $I_{0}(\lambda )$ in Eq. (2). As a result, we could derive the 14-band spectral transmittance as $I_{1}(\lambda )/I_{0}(\lambda )$, where the center wavelengths ranged from 440 to 700 nm at 20 nm intervals. Then, we compared the measured values with the nominal transmittance data provided with the color chart slide, as shown in Fig. 1(b). From the comparison result, we confirmed that the spectral transmittance of the color patcher was measured with good approximation from 440 to 700 nm at 20-nm intervals. For visual evaluation, we obtained a color image from a captured MS image. Specifically, we converted an MS image to sRGB color image data with the sRGB standard^24^ and CIE 1931 color matching functions. The color of the generated sRGB color image was visually equivalent to the color image obtained by a whole slide scanner. The color reproduction in this manner is not highly accurate but acceptable here because accurate color reproduction is not the objective of this study.

### 3.2 Estimation of Stain Matrix and Normalization of Estimated Dye Amount

**Fig. 3:**
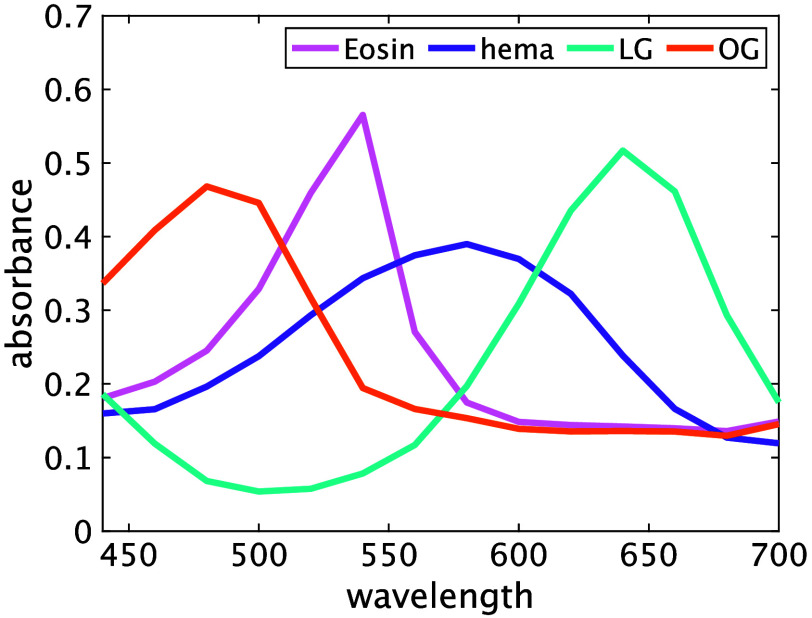
Spectral absorbance of Papanicolaou stain.

As mentioned in Sec. 2.4, we used single-stain samples to determine the stain matrix H required for spectral unmixing. The specimens were gathered from a uterine cervix and an oral cavity. Table 1 shows the number of cases and MS images. Some captured images are shown in Fig. 2, where MS images were transformed to sRGB images using the CIE 1931 color matching functions and sRGB standard, as mentioned in the previous section. The lipid stained with BY is hard to observe in Fig. 2, and the lipid detection is less crucial in the observation of mucinous cytoplasm, which is the primary focus of our work. Thus, we considered only EY, H, LG, and OG dyes in the following experiment. We cropped five or six samples of 5×5  pixels from each MS image, calculated 25 absorbances from Eq. (2), and averaged and normalized them. Figure 3 shows the normalized absorbance of EY, H, LG, and OG, which correspond to $\varepsilon_{l}(\lambda )$. The spectral absorbance of H has a broad peak between 550 and 600 nm, which represents a blue to purple color. EY has a sharp peak in the absorption spectrum between 500 and 550 nm, representing pink; the spectral absorbance of LG is very low between 480 and 540 nm, which shows a light blue-green; and OG absorbs spectrum near 480 nm, mostly transmits the wavelengths longer than 530 nm, and represents an orange. The spectral absorbance curves in Fig. 3 reflect the characteristics of each the corresponding dyes in the Papanicolaou stain. As a result, we obtained the spectral absorption coefficient matrix H, and c was estimated using the pseudo-inverse matrix of H.

**Table 1:** Number of the single-stain MS images.

	Cases	MS image				
		H	EY	LG	OG	BY
Uterine cervix	3	18	21	17	19	18
Oral cavity	1	7	6	6	6	6

Next, the reference values for normalizing the dye amounts were determined from Papanicolaou-stained samples, as explained in Sec. 2.7. We selected seven well-stained areas for each dye and analyzed the histogram of the estimated dye amount. Then, the 99 percentile values $Q_{l}\;(l\in \{\mathrm{EY},\;H,\;\mathrm{LG},\;\mathrm{OG}\})$ were set as the reference value for each dye amount, and $c_{l}$ was divided by the reference value to obtain the normalized dye amount. The reference values were $Q_{\mathrm{EY}}=2.003$, $Q_{H}=1.889$, $Q_{\mathrm{LG}}=2.550$, $Q_{\mathrm{OG}}=1.213$.

### 3.3 Experimental Verification of Dye Amount Estimation

**Fig. 4:**
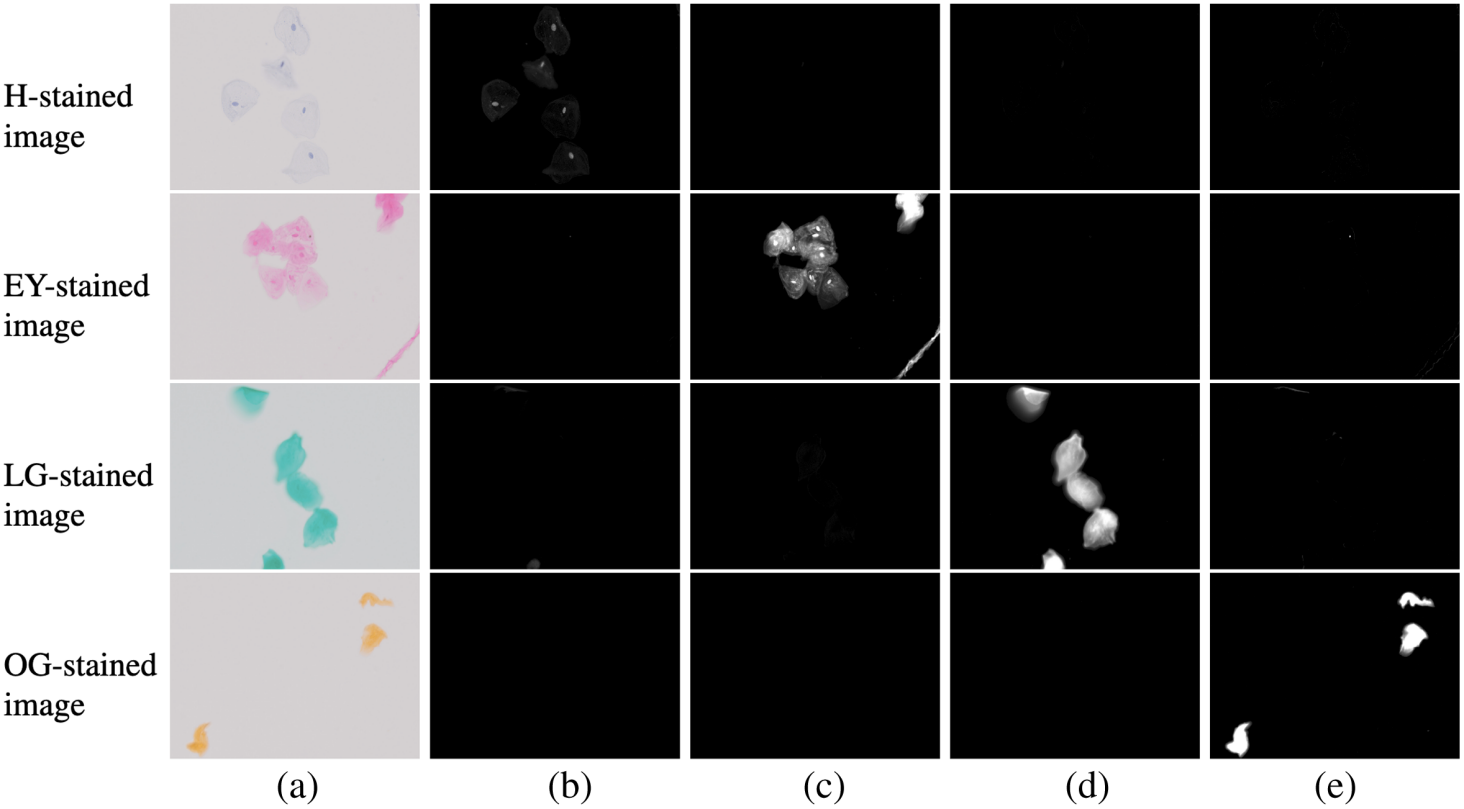
Estimated dye amount maps of single-stain images using the proposed method. (a) RGB image. (b) Results of H. (c) Results of EY. (d) Results of LG. (e) Results of OG.

First, we applied the proposed method to single-stain images and verified the dye amount estimation performance of the proposed method. The reason for using single-stain images for the verification is that obtaining a ground truth is challenging. We used the images of oral cells stained with H, EY, LG, and OG, with six images for each dye. We show the average and the variance of the estimated dye amount and some dye amount maps in Table 2 and Fig. 4, respectively. In Table 2, we calculate the dye amount by averaging it in the region where any dye is nonzero. We transform MS images to sRGB images in Fig. 4. Table 2 and Fig. 4 show that a single-stain dye amount image was estimated in each corresponding dye component, and other dye abundance images were almost 0. This means that the proposed method achieves high estimation performance for all dyes. Even though not very noticeable, the deeply stained area had subtle amounts of other dyes, e.g., the dye amounts of H and OG were nonzero in the center bottom and top in the LG-stained image. Note that the average value of other dye abundance images in Table 2 is very low even though there are these areas because it was averaged in the region where any dye is nonzero. The pixel values of the long-wavelength region (640 to 650 nm) were very small in the area strongly stained with LG, and the spectrum obtained by the MS image was distorted, probably inducing the error in unmixing.

**Table 2:** Average of the dye amount using the proposed method from single-stain images. The bold font is shown as the highest dye amount in each observation.

Observation	Dye			
	H	EY	LG	OG
H-stained image	**0.1075**	$1.908\times 10^{-4}$	$2.223\times 10^{-3}$	$4.225\times 10^{-3}$
EY-stained image	$4.871\times 10^{-4}$	**0.2785**	$2.355\times 10^{-4}$	$2.164\times 10^{-3}$
LG-stained image	$5.861\times 10^{-3}$	$6.441\times 10^{-3}$	**0.4867**	$4.349\times 10^{-3}$
OG-stained image	$1.362\times 10^{-4}$	$2.540\times 10^{-4}$	$1.506\times 10^{-4}$	**0.8493**

**Fig. 5:**
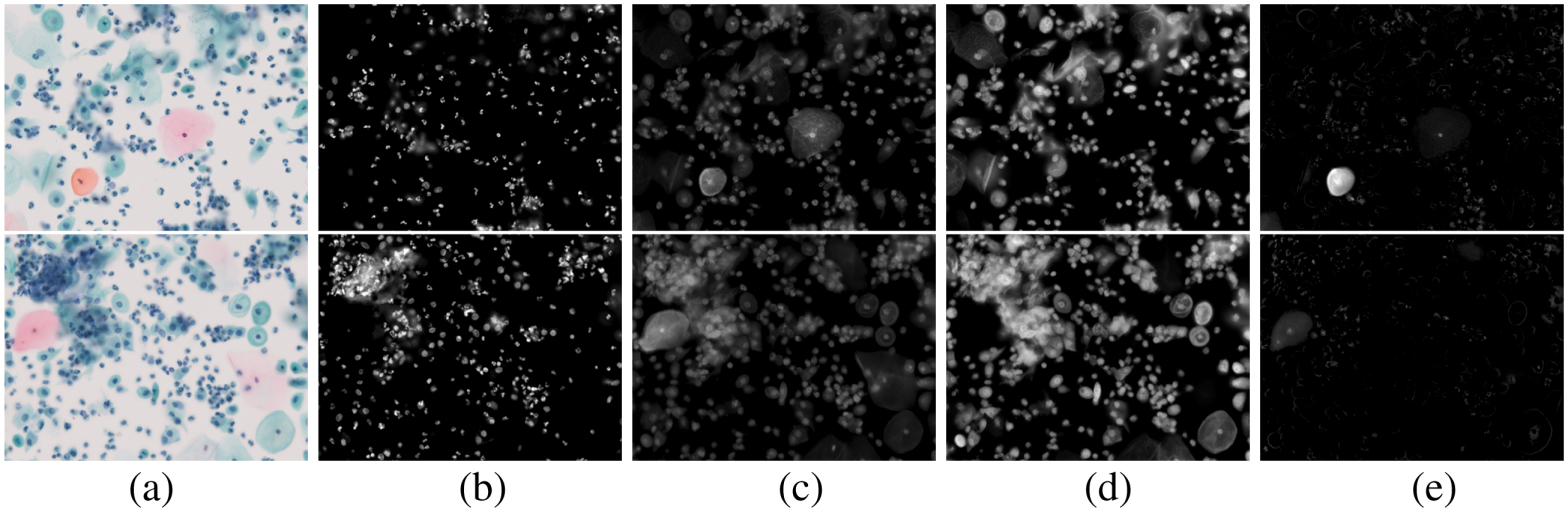
Estimated dye amount maps from Papanicolaou-stained image using the proposed method. (a) RGB image. (b) Results of H. (c) Results of EY. (d) Results of LG. (e) Results of OG.

Next, the proposed method was applied to Papanicolaou-stained images, and Fig. 5 shows two examples of the estimated results. The dye amount maps estimated using the proposed method are visually reasonable. For example, almost all cell nuclei contain H. The EY, LG, and OG dye amount maps show that each dye exists in the cytoplasmic regions that appear light green, pink, and orange, respectively. In contrast, the EY, LG, and OG dye amount maps also show some cell nuclei, which is attributable to two reasons: co-stain and unmixing error. In the single-stain RGB images of Fig. 4(a), the color of the nuclei is slightly darker than that of the surrounding cytoplasm in EY and LG. Even though the nuclear stain by H inhibits staining with other dyes, some EY and LG may retain the nuclei stain. The unmixing error of the H-stained image is observed weakly in Figs. 4(c)–4(e). We can find faint nonzero pixels in EY, LG, and OG images of the H-only single-stain case. In the densely stained pixels, the pixel values approach 0, and the quantization error and the dark current noise affect the unmixing accuracy. Another cause of the unmixing error may be the inaccuracy in characterizing the multispectral imaging system, e.g., the bandwidth in the spectral sensitivity is neglected in the model described in Sec. 3.1.

Figure 5 also shows that the cytoplasm areas exhibit light green, pink, and orange colors, respectively characterized by LG, EY, and OG dyes and their mixture. Different dyes stain the cytoplasm depending on molecular weight, diffusibility, and differentiation. If the cytoplasm has the characteristics between LG and EY or EY and OG, the cytoplasm may be stained with both dyes.

### 3.4 Quantification of Dye Amounts in Cytoplasmic Mucin of EC and LEGH Cells

As an application of the proposed estimation in a Papanicolaou-stained specimen, we analyzed the estimated dye amounts in the cytoplasmic mucin of EC and LEGH cells. In the experiment, we captured MS images of EC or LEGH cells: the numbers of MS images containing EC and LEGH were 21 and 25, respectively. The specimens were gathered from four patients.

**Fig. 6:**
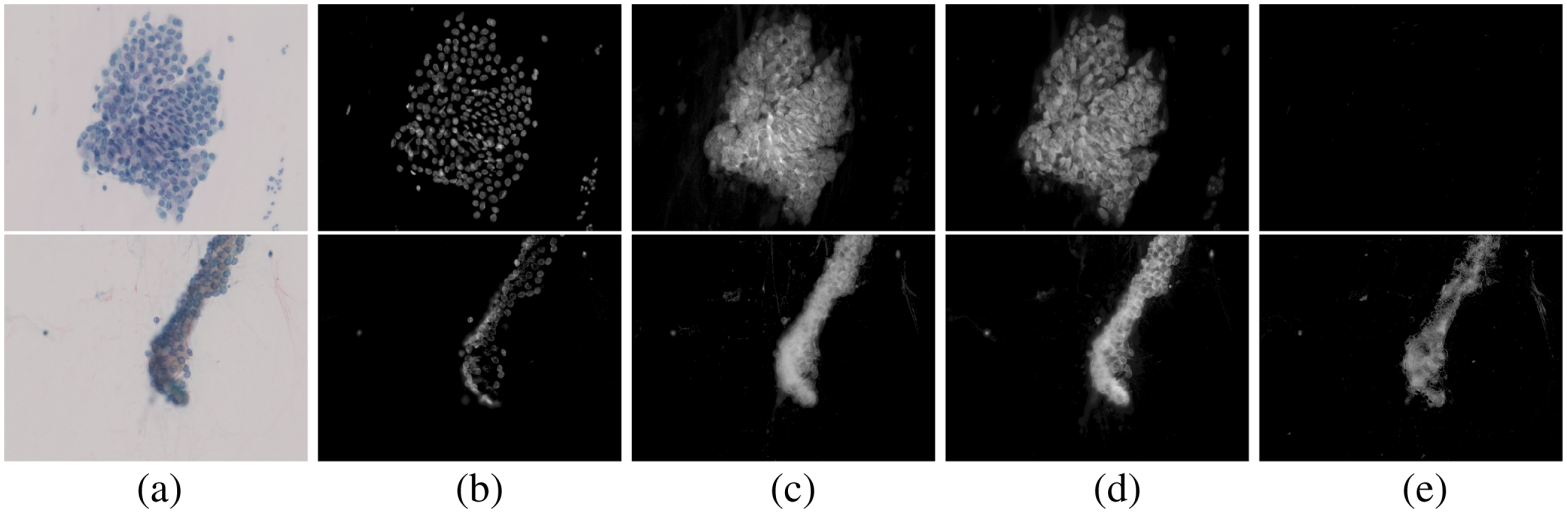
Estimated results of EC (top) and LEGH (bottom) images using the proposed method. (a) RGB image. (b) Results of H. (c) Results of EY. (d) Results of LG. (e) Results of OG.

Some results of the proposed estimation method using MS images are shown in Fig. 6. The RGB images indicate that the mucin of EC is pinkish and that of LEGH is orangish, as reported in Refs. 5 and 6. The dye amount maps shown in Figs. 6(b)–6(e) clearly exhibit that the cytoplasmic mucin in LEGH cells is stained with OG, whereas EC cells contain no OG.

**Fig. 7:**
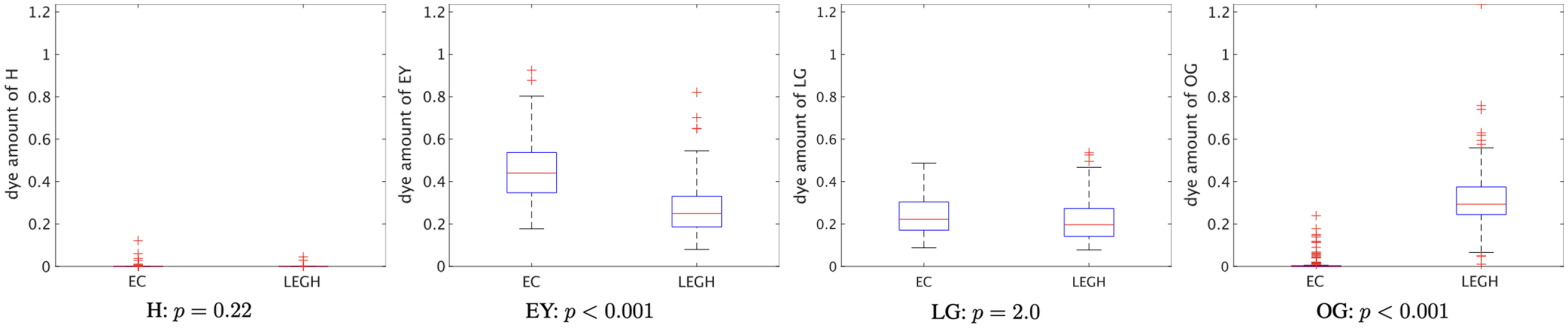
Box plot of the average dye amount with each p-value using Mann–Whitney U test.

For quantitative analysis of the difference in dyes, we extracted the mucin samples in 10×10  pixels from the cytoplasmic regions in the MS images and calculated the average dye amount in the area where any dye exists. The numbers of EC and LEGH mucin samples were 124 and 120, respectively. The Mann–Whitney U test, a nonparametric test, was employed to see whether there were significant differences between the LEGH and EC cells. The box plot of each dye with their p-value is shown in Fig. 7. It can be confirmed that there is a significant difference in the EY and OG cases (p<0.001). The plots show that none of the samples include H because we chose the cytoplasmic mucin area. As a result, quantitative analysis of each dye amount is a utility for LEGH mucin detection, and the method plays an essential role in this analysis.

### 3.5 Classification of LEGH and EC Cells Based on the Estimated Dye Amount

**Fig. 8:**
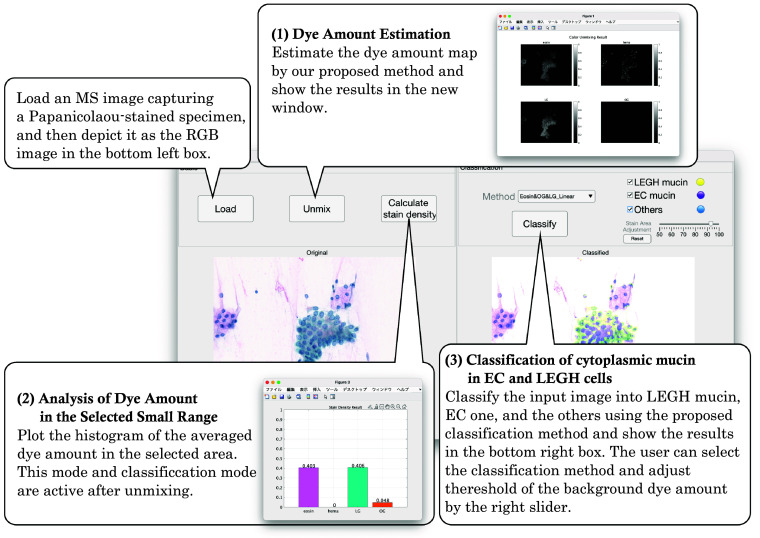
Overview of the proposed application software tool.

We developed a system to experimentally examine the classification of the LEGH cells based on the quantification of the dye amount in cytoplasmic mucin using the proposed method. Figure 8 shows the main user interface screen of the developed system. This system consists of three functional components: dye amount estimation, analysis of dye amount in the selected small range, and classification of EC and LEGH mucin. The classification is performed in the following three steps:

(1) Dye amount estimation

**Fig. 9:**
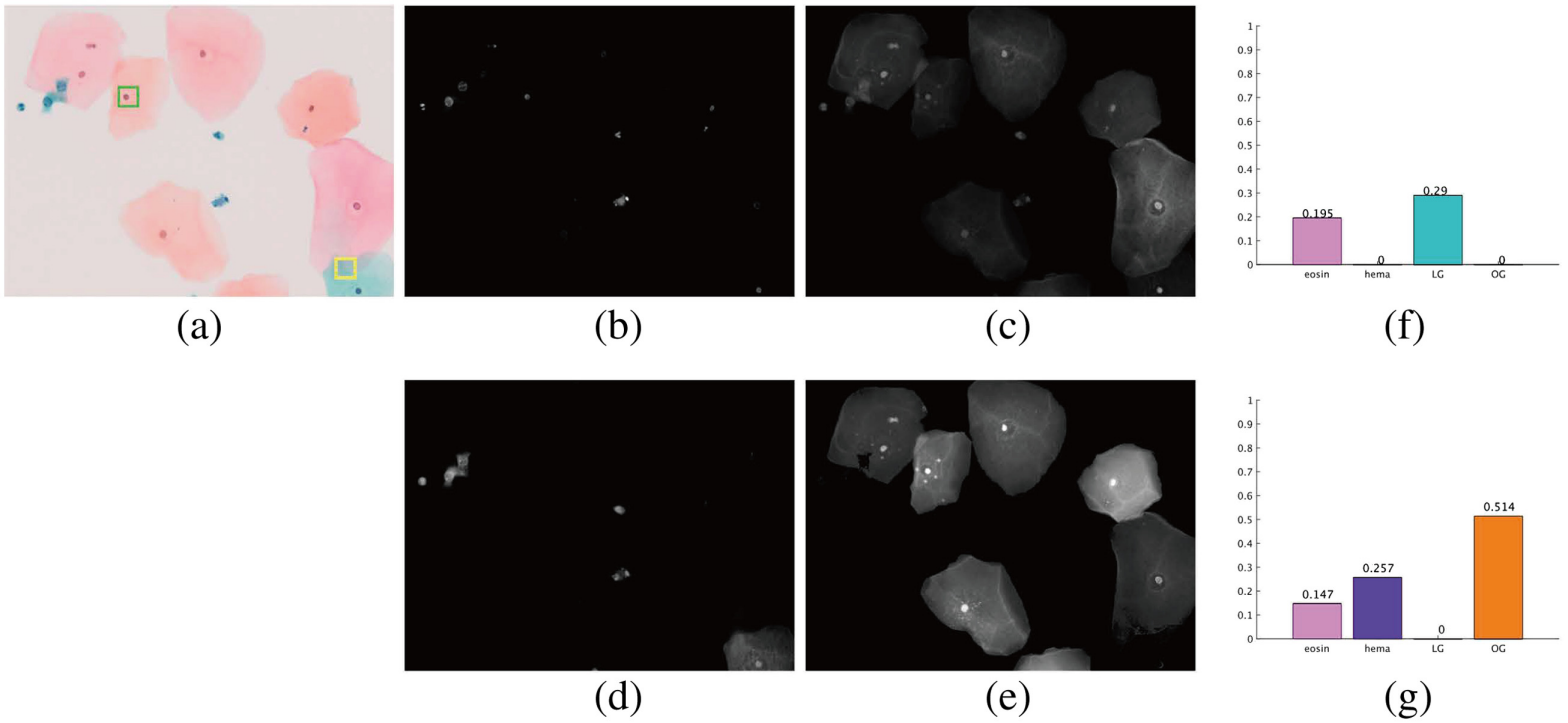
Results on the proposed application software tool. (a) Input image. (b) H map. (c) EY map. (d) LG map. (e) OG map. (f) Dye amounts in the yellow-bordered area of panel (a). (g) Dye amounts in the green-bordered area of panel (a).

By unmixing an MS image of a Papanicolaou-stained specimen, the dye amount maps are estimated. A user selects the background area from the depicted RGB image to derive the thresholds $T_{l}$ in Sec. 2.6. The obtained dye amount maps are shown in Figs. 9(b)–9(e).

(2) Analysis of dye amount in the selected small range

If a user selects a small rectangular area for analysis, the system shows the average dye amounts to H, EY, LG, and OG in the selected area. Figures 9(f) and 9(g) depict the bar graph of the dye amount in the selected yellow- and green-bordered area of panel (a), respectively. The yellow-bordered area includes the cytoplasm of two overlapping cells stained with LG and E. In the green-bordered area, we select a nucleus with cytoplasm around it, and the bar graph reveals H that stains the nucleus and EY and OG that stain the cytoplasm. The cytoplasm appears to be a mixture of orange and pink, and the graph shows a considerable amount of OG and less EY stain in this region. Although it is difficult to visually assess whether EY stains the cytoplasm or the ratio of EY and OG from the RGB image, the dye amounts obtained from the image provide quantitative information that would help reveal the characteristics of cells in Papanicolaou staining.

(3) Classification of cytoplasmic mucin in EC and LEGH cells

**Fig. 10:**
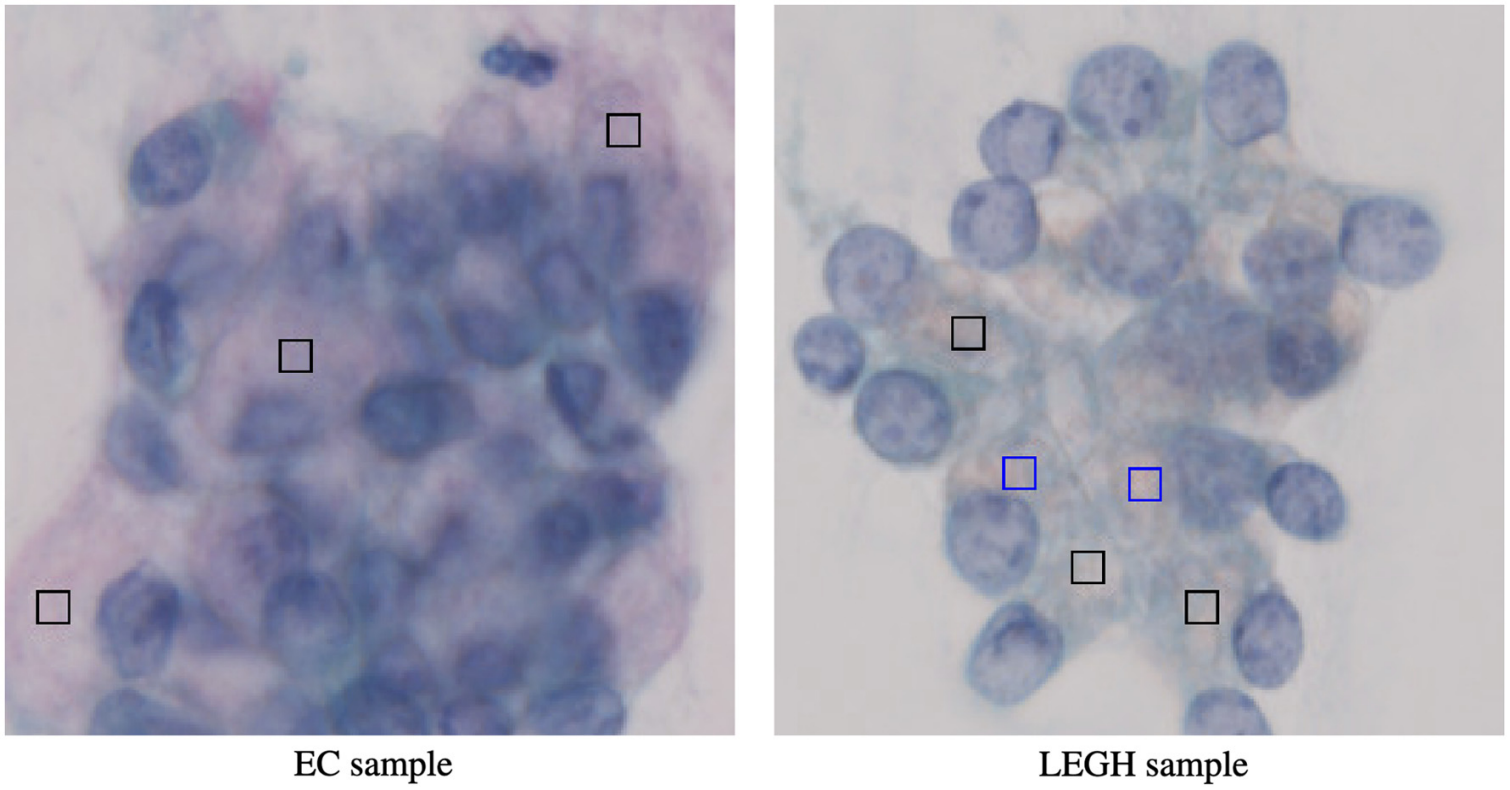
Samples of EC and LEGH mucin (black frames).

**Fig. 11:**
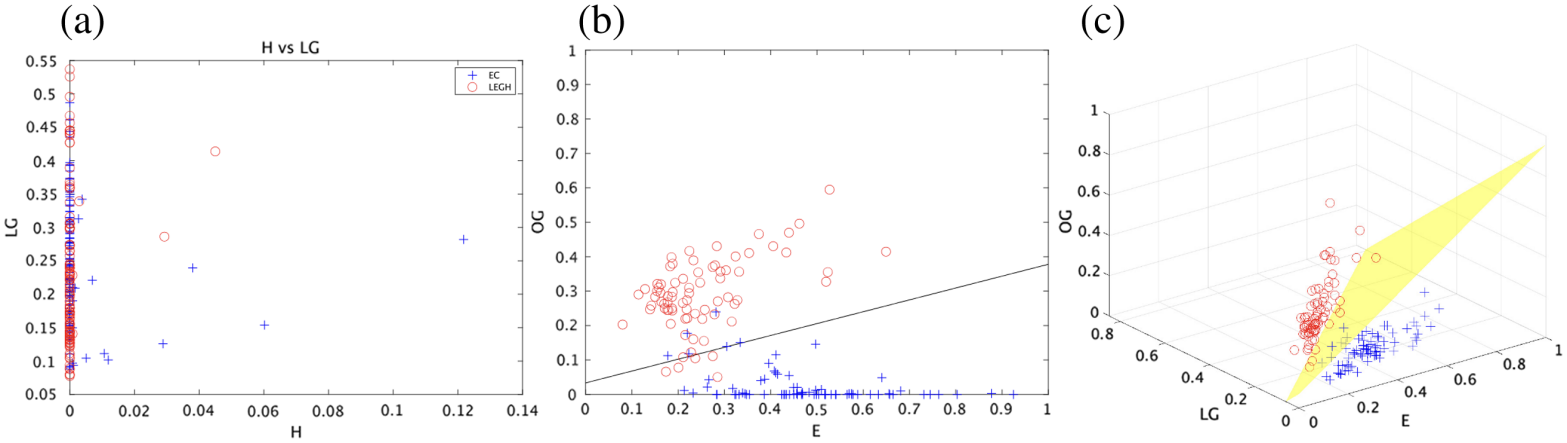
Samples on 2D H-LG plane (a), 2D EY-OG plane with the decision boundary of 2D-Fischer (b), and 3D EY-LG-OG space with the decision boundary of 3D-Fischer (c).

The experiment of classifying EC and LEGH cells was performed using the method explained in Sec. 2.7. The samples were 10×10  pixels cropped from the images of two EC cases and two LEGH cases, and the numbers of samples were 75 and 76, respectively. Here, Fig. 10 shows some samples for the classification. In Fig. 11, the graphs plot all dye amounts averaged in each sample in the H-LG and EY-OG planes and the EY-LG-OG space. As the dye amount of H is almost zero and useless for classification, the linear discriminant functions D(c) were derived from the samples in the 2D and 3D cases (named 2D-Fischer and 3D-Fischer, respectively), as follows:

$$2D\text{-}\text{Fischer}:\;D_{2D}(c)=15.01\mathrm{EY}-43.55\mathrm{OG}+1.454,\;3D\text{-}\text{Fischer}:\;D_{3D}(c)=41.52\mathrm{EY}-55.34\mathrm{LG}-46.79\mathrm{OG}+3.263,$$ (5)

where EY, LG, and OG are the averaged amount of each dye. The decision boundary between EC and LEGH is given by D(c)=0, namely, the cytoplasm is classified into LEGH if D(c)≥0 and EC if D(c)<0.

We verified the performance of the classification using samples obtained by averaging 10×10  pixels cropped from another case: 49 EC and 44 LEGH samples (called test samples). For comparison, we adopted a method, which decides the decision boundary using the RGB values. This is because there is no LEGH detection method for Papanicolaou-stained specimens, and some methods^7^^,^^8^ quantify the color of the specimens by RGB. In our experiment, we calculated sRGB values from the spectral data. The performance is higher than the practical RGB-based imaging system because the sRGB values do not involve color variation due to the device dependence. The linear discriminant function is as follows:

$$D_{\mathrm{sRGB}}(c)=83.41R-368.9\;G+365.8B-74.22.$$

The confusion matrix and the four quality measures of the results are shown in Tables 3 and 4, respectively, where we adopted accuracy, precision, recall, and f value to evaluate the performance. The tables show that our proposed classifiers effectively classify EC and LEGH mucin with high accuracy and are slightly superior to the compared method.

**Table 3:** Confusion matrix on the test samples.

label	Pred.					
	sRGB		2D-Fischer		3D-Fischer	
	EC	LEGH	EC	LEGH	EC	LEGH
EC	39	10	49	0	44	5
LEGH	0	44	5	39	0	44

**Table 4:** Four quality measures of the results on the test samples. The bold font is shown as the highest value.

Measures	Boundary		
	sRGB	2D-Fischer	3D-Fischer
Accuracy	0.8925	**0.9462**	**0.9462**
Precision	**1**	0.8864	**1**
Recall	0.8148	**1**	0.8980
F value	0.8980	0.9398	**0.9462**

**Fig. 12:**
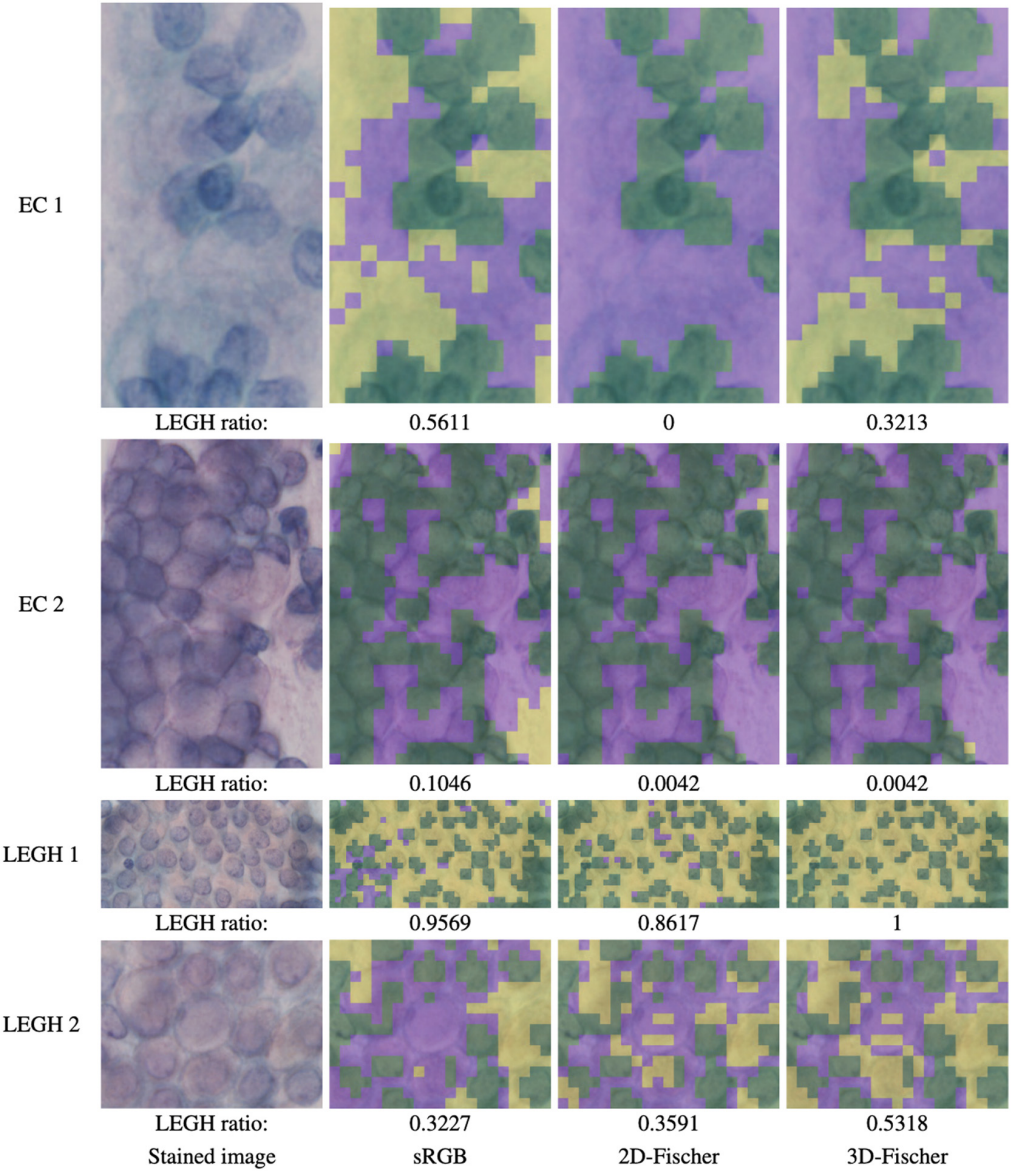
Classification results by the compared method, 2D-Fischer, and 3D-Fischer (purple: EC, yellow: LEGH, green: background and cell nuclear).

Moreover, we experimented with the classification of cell clusters. The EC or LEGH cell cluster images (ranging from 126×72 to 941×659  pixels) were cropped from the MS images. The number of the images was 55 (EC: 25 and LEGH: 30). The classification process is depicted in Sec. 2.7. In addition, we calculated the ratio of the patches classified into LEGH to all patches, excluding nuclear and background patches. Figure 12 shows some of the classification results. In EC cases, 2D-Fischer effectively classifies EC mucin. 3D-Fischer misidentifies on the left bottom of EC 1. These areas are greenish, so there would be a few EC mucin. For sRGB cases, a few stained areas are classified into LEGH. In the images of LEGH 2 in Fig. 12, as there is a pinkish and orangish area, patches are classified into EC in the sRGB case.

**Fig. 13:**
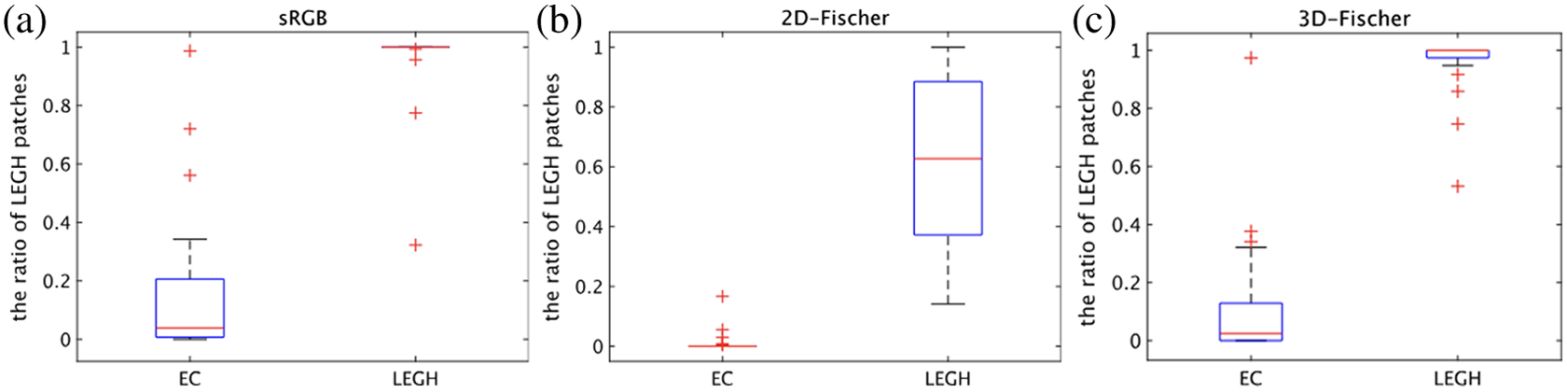
Boxplot of the ratio of LEGH patches [(a) sRGB, (b) 2D-Fischer, (c) 3D-Fischer].

Figure 13 depicts the boxplots of the LEGH ratio of the 55 cell-cluster images. The figure shows that the compared method and 3D-Fischer can effectively classify EC and LEGH cell clusters. In particular, in 3D-Fischer, many EC and LEGH cell clusters have LEGH ratios near 0 and 1, respectively. The above results show that 3D-Fischer is slightly superior to the other methods.

## 4 Discussion

From the above experimental results, one can see the performance of our dye amount estimation method and the utility of the dye amount analysis for Papanicolaou-stained specimens. However, this study has two discussion points: the suitable band number and the relationship between the thickness of the specimen and the dye intensity.

In this paper, we used Vectra^®^3 as an MS microscope, which provides a 14-band MS image. Even though 14 bands are exaggerated and redundant for this task, we used all 14 bands as a basic experiment confirming the spectral absorbance curves of four different dyes, as shown in Fig. 3. Theoretically, four narrow bands are sufficient, and the wavelengths are appropriately selected because the dye amount estimation problem should be well posed for the observation with four or more bands. However, we verified that the estimated performance from the four-band observation is not sufficient. This inaccuracy would be caused by the systematic error in the spectral characterization of the MS microscope. The spectral sensitivity of the 14-band MS camera is not rigorously narrow, and it is difficult to measure the spectral sensitivity of the commercial MS microscope. Instead, we confirmed that the spectral transmittance measurement was almost acceptable using a color chart slide, as shown in Fig. 1. Moreover, it is noteworthy in this paper to demonstrate that the off-the-shelf MS imaging device can be applied for spectral unmixing to quantify the dye amounts. However, the error in the approximation of Eq. (3) is not negligible. When we use a sufficient number of bands, the dye amount estimation could be more robust. In the future, we shall confirm the suitable band number.

The thickness of cytological specimens often affects the image analysis in digital cytology. In the proposed method, the observed dye amount depends on the thickness of an area. We used an LBC and a conventional specimen; the latter, our main used type, is generally known to be thicker. As we try to interpret the color observed under a microscope by the utility of spectral unmixing in this work, the image was captured specimens by manually adjusting the focus on the cells of interest with one layer. If the extended focus is used, more dye will be observed in thicker cells as the absorbance of different depths is integrated. On the other hand, in the case of a single-plane MS image, it primarily represents the dye amount within the depth of field. Out-of-focused objects are blurred but may affect the dye amount if they remain visible. If we observe a z-stack, we can quantify the sum of dye amounts of visible objects at each depth. The color of a pixel becomes a mixture of different dyes if two cells overlap, as shown in the yellow-bordered area in Fig. 9(a). The dye amount indicates that the region contains EY and LG because the squamous cells stained with EY and LG are overlapped. These two cells are clearly separated in the dye amount map. Although the analysis of the relationship between thickness and estimated performance using z-stack or extended focus needs to be addressed in the future, we believe that dye amount estimation in the single-layer image offers a significant and valuable approach for Papanicolaou-stained specimens.

## 5 Conclusion

This paper proposes a new image analysis method for the Papanicolaou-stained specimen using an MS observation, which estimates the dye amount map of H, EY, LG, and OG stains and utilizes the estimated dye amounts for differentiating the cytoplasmic mucin of EC and LEGH cells based on quantitative analysis. To verify the estimation performance, we experimentally apply the proposed method to MS images capturing single-stained and Papanicolaou-stained specimens. As a result, our proposed method can effectively estimate each dye amount. Moreover, we analyze the characteristics of dye amounts in EC and LEGH cells and elucidate that the yellowish appearance of the cytoplasmic mucin in LEGH cells originates from more OG and less EY. Based on the quantified dye amount, the EC and LEGH cell clusters can be classified, showing the possibility of its application to a diagnosis support system with our proposed method in Papanicolaou-stained cytological specimens. Nevertheless, there are several issues for a practical application: a method for determining the stain matrix without relying on single-stain samples, utilizing z-stack or extended focus features, and experimental validation when the color variation in the Papanicolaou-stain is not negligible. In the future, it is expected to construct an LEGH diagnosis supporting system utilizing both the morphological characteristics of nuclei expressed in Ref. 4 and the color characteristics of cytoplasm with a Papanicolaou stain revealed in this work.

## Data Availability

We shared the code of the proposed method on https://github.com/SaoriTakeyama/Pap_DAEst. The data are not publicly available.
